# The *pugilist^Dominant^* Mutation of *Drosophila melanogaster*: A Simple-Sequence Repeat Disorder Reveals Localized Transport in the Eye

**DOI:** 10.1371/journal.pone.0151377

**Published:** 2016-03-21

**Authors:** Yikang S. Rong, Mary M. Golic, Kent G. Golic

**Affiliations:** Department of Biology, University of Utah, Salt Lake City, Utah, United States of America; University of Valencia, SPAIN

## Abstract

The *pugilist-Dominant* mutation results from fusion of a portion of the gene encoding the tri-functional Methylene Tetrahydrofolate Dehydrogenase (E.C.1.5.1.5, E.C.3.5.4.9, E.C.6.3.4.3) to approximately one kb of a heterochromatic satellite repeat. Expression of this fusion gene results in an unusual ring pattern of pigmentation around the eye. We carried out experiments to determine the mechanism for this pattern. By using FLP-mediated DNA mobilization to place different *pug*^*D*^ transgenes at pre-selected sites we found that variation in repeat length makes a strong contribution to variability of the pug phenotype. This variation is manifest primarily as differences in the thickness of the pigmented ring. We show that similar phenotypic variation can also be achieved by changing gene copy number. We found that the *pug*^*D*^ pattern is not controlled by *wingless*, which is normally expressed in a similar ring pattern. Finally, we found that physical injury to a *pug*^*D*^ eye can lead to pigment deposition in parts of the eye that would not have been pigmented in the absence of injury. Our results are consistent with a model in which a metabolite vital for pigment formation is imported from the periphery of the eye, and *pug*^*D*^ limits the extent of its transport towards the center of the eye, thus revealing the existence of a hitherto unknown mechanism of localized transport in the eye.

## Introduction

The *pugilist* (*pug*) gene of *Drosophila melanogaster* encodes an enzyme with three activities in tetrahydrofolate metabolism. Null mutations of *pug* cause a subtle, transitory and recessive reduction in pteridine pigmentation in the eyes of newly-eclosed flies. The dominant *pug*^D^ mutation reduces pigment throughout the eye, with an effect that is especially noticeable in a background where only pteridines are present, e.g. *vermillion* (*v*). The eyes of such flies (*v; pug*^*D*^*/+*) exhibit an unusual ring of pigmentation around the periphery of the eye and a few scattered spots of pigment in the center of the eye, but are otherwise completely white-eyed. Ommochrome pigmentation is also affected by *pug*^*D*^, but to a lesser degree [1]. The protein encoded by *pug* has also been implicated in the response to Parkinson's disease, aging and oxidative stress, immunity [2–6].

The *pug*^*D*^ mutation was created at one of the junctions of an X-ray induced inversion on chromosome *3*. The mutant allele consists of three parts assembled from three different genomic locations. The upstream regulatory elements and the first approximately one-third of the coding region, including the start codon, are derived from the *pug* gene, which normally encodes the tri-functional methylenetetrahydrofolate dehydrogenase (MTHFD, located at 86C) and generates products that participate in 1-carbon transfer reactions. This activity is prominently involved in purine synthesis, and since pteridine biosynthesis is based on GTP, this provides one possible connection between *pug*^*D*^ and pigment deposition (reviewed [7]). The portion of the gene that remains in the *pug*^*D*^ allele should encode approximately two-thirds of the methylene tetrahydrofolate dehydrogenase and cyclohydrolase domain [8]. It lacks the ATP-binding site and the entire formyltetrahydrofolate synthetase domain. It seems unlikely that *pug*^*D*^ retains any of its normal enzymatic functions. The second *pug*^*D*^ coding segment is approximately one kb of highly repetitive sequence, consisting almost entirely of iterations of the satellite sequence AGAGAGA (although oriented with TCTCTCT on the sense strand in *pug*^*D*^). These almost certainly derive from centric heterochromatin where such repeats are abundant [9], and were captured at the inversion junction during repair of the breakpoint that generated the inversion. Transcription and translation of both coding segments is necessary for the mutant phenotype [1]. The translated repeats constitute about two-thirds of the pug^D^ protein. The third portion consists of *rab7* oriented in the opposite direction, and does not contribute to the *pug*^*D*^ phenotype.

Because *pug*^*D*^ does not simply reproduce the phenotype of a *pug* null allele, we consider *pug*^*D*^ to be a gain-of-function, rather than loss-of-function allele. The strong phenotype produced by *pug*^*D*^ relative to *pug*-*null* alleles, even in the presence of two copies of *pug*^*+*^, also indicates that it is not merely an antimorph. For these reasons, we consider the *pug*^D^ mutation to be neomorphic. This does not exclude the possibility that it also interferes with the action of *pug*^*+*^, but the Pug^D^ protein is clearly doing more than that.

The ring pattern of pigmentation produced by *pug*^*D*^ is certainly the most unusual aspect of its pug^D^ phenotype. A very limited number of mutations have been identified that produce a ring or partial ring pattern of pigmentation. The cause for such patterns has not been identified in any of these cases. In this study we explore the genesis of ring pigment patterns in *pug*^*D*^. We have observed that *pug*^*D*^ transgenes show a large degree of phenotypic variation, primarily in the thickness of the pigmented ring. The phenotypes range from those that are essentially identical to the original *pug*^*D*^, to weak alleles that are indistinguishable from wildtype [1]. The work we report here was carried out to determine the basis for the phenotypic variation, and to investigate models for the ring pattern of pigmentation.

## Materials and Methods

### Fly Stocks

Mutations and chromosomes not described here are described by Lindsley and Zimm [10]. Flies carrying a *GMR-wg* construct were provided by Andrew Tomlinson [11]. Flies carrying the *Gla*^*1*^ mutation were provided by Konrad Basler [12]. Flies with the construct p*P[w*^*+*^, *GMRP35]* were provided by Bruce Hay [13]. Flies with the construct p*P[sev-wg]* were provided by Kenneth Cadigan [14].

### *Glazed* and *GMR*-wg Crosses

To assess the effect of ubiquitous *wingless* expression in the eye we crossed *v; pug*^*D*^*/TM6* females to either *GMR-wg/TM7* males, to *Gla*^*1*^ *Bc bw/CyO* males [12] or to *+/Gla*^*1*^ *Bc Elp* males (in which the + homolog carried an unidentified deficiency). The *v* sons with the desired *wg* allele were examined for pigmentation.

### Eye Injection Experiments

Pupae of *v; pug*^*D*^ */TM3*, *Ser* were picked at stages ranging from 0 to 2 days into pupation. They were mounted on slides with double stick tape. A glass needle similar to that used for embryo injections was employed to inject solutions into the eyes of these pupae. Injected pupae were left on slides placed on moist paper. The solutions injected were: (1) 0.85 mM guanosine in pH2.0 HCl solution (guanosine); (2) pH2.0 HCl solution (HCl); (3) 0.5X PBS solution (PBS).

### *pug*^*D*^ Transgenes

The construction of *pug*^*D*^ transgenes in the vector *P[X97]* has been previously described [1]. The construct used for most experiments reported here carried the 3.4 kb *Kpn*I—*Bam*HI *pug*^*D*^ genomic fragment. This fragment contains only the promoter for the two short transcripts identified for this gene (pug-RA, pug-RC), and contains no coding sequences from the adjacent gene CG14863 (Flybase: [15]). The *pug*^*S*^ gene was a derivative of the original *pug*^*D*^ clone in which the AGAGAGA repeats were spontaneously reduced to 300 bp during overnight bacterial culturing.

### *P* Element Transposition Screen

The *X* chromosome insertion 1A carries the *P* element construct of p*P[X97*, *pug*^*D*^*]* which has the *v*^*+*^ marker [1]. Females of the genotype *v P[X97*, *pug*^*D*^*]1A; ry* were crossed to *w*^*1118*^*; Sb Δ2–3 (99B)/TM6*, *Ubx* males, which carry the *Δ2–3 (99B)* insertion to provide P transposase [16]. Individual *v P[X97*, *pug*^*D*^*]1A; Sb Δ2–3 (99B)/ry* males were mated to two or three *v; ry* females. *Sb*^*+*^ male offspring that are *v*^*+*^ (with or without any pigmentation defect) were retained. From the offspring of a single male, one fertile male was kept among the *v*^*+*^ males with the same pigmentation pattern. This ensured that only males with independent *P* transposition events to autosomes were retained. Lines were established from these males. Each line was assigned with a number. All the v^+^ lines with normal pigmentation were later discarded except three: 46A, 50 and 74. All the lines were mapped by segregation from dominantly marked autosomes. No insertion on chromosome *4* or *Y* was recovered.

### FLP-Mediated DNA Mobilization

Mobilization experiments were done as described [17]. Briefly, for insertions on chromosome *3*, females that were *w*^*1118*^ *P[ry*^*+*^, *70FLP]3F; P[RS3r]/TM6* were mated to *v; P[v*^*+*^*]/TM6* males, with 4–5 pairs per vial. *P[RS3r]* is the *FRT*-bearing target site element that carries the 3’ portion of the *white* gene, and P*[v*^*+*^*]* represents the *P[X97]* element carrying a derivative of the *pug*^*D*^ gene and a functional 5’ portion of *white* (along with a non-functional 3’ portion of *white*). Parents were transferred every 2 days. The vials were heat shocked for one hour at 37˚C immediately after each transfer. Male progeny that were *w*^*1118*^ *P[ry*^*+*^, *70FLP]3F; P[RS3r]/P[v*^*+*^*]* were mated to *w*^*1118*^ females with one male and 2–3 females per vial. Progeny of this cross exhibiting eye pigment, indicating that a *w*^*+*^ gene had been reconstructed by integration of the *pug-*bearing extrachromosomal circle at the target site, were retained. For donor insertions on chromosome *2*, individual *w*^*1118*^ *P[ry*^*+*^, *ß2tFLP]; P[v*^*+*^*]/+; P[RS3r]/+* males were mated to 2–3 *w*^*1118*^ females per male. Lines were established from pigmented progeny.

For each *RS3r* target site, one of the recovered integration events was tested to confirm that it was the result of FLP-mediated mobilization to the target *FRT*. First, we simply outcrossed heterozygous females to ensure that *pug*^*D*^ and *w*^*+*^ could not be separated by recombination. We found no cases in which this occurred. A second test was based on the fact that *pug*^*D*^ and part of *w*^*+*^ are flanked by two direct *FRT*s after donor integration. When FLP is expressed in the soma, by crossing to flies carrying the heat shock inducible *70FLP* gene and heat-shocking offspring during third instar, mosaicism for both *pug*^*D*^ and *w*^*+*^ should be observed. Adult eyes were scored for *pug*^*D*^ mosaicism in a *v* background, or for *white* mosaicism in a *w* background. In previous work [17], only 1 of 46 events recovered by the scheme we used here was not specifically targeted, and that one event did not produce white mosaicism with FLP. In the present work, all the tested lines exhibited mosaicism for both phenotypes in response to FLP expression, supporting the conclusion that they resulted from FLP-mediated mobilization to the target *FRT*. Each mobilized *pug*^*D*^ gene was named for both the donor insertion and the target site. For example, the *pug4A-2* transgene was the result of mobilizing the *pug*^*D*^ gene in donor line 4A to the target site of *RS3r-2*.

### Characterization of AGAGAGA Repeat Length

A combination of Southern blot and PCR analyses were done to determine the genomic structure of the *pug*^*D*^ genes mobilized to *RS3r-2*. Genomic DNA was isolated as described [1]. Southern blot and PCR analyses were performed according to standard protocols. The probe was labeled and detected using the Genius non-radioactive kit (Roche). The exposed film was scanned, and the gel was straightened electronically in Photoshop for analysis. PCR was used to amplify regions of *pug*^D^ upstream and downstream of the AGAGAGA repeats to determine their lengths. The primers used in this work were: 1, w14178d (5’-TGTGTGTTTGGCCGAAGTAT-3’); 2, TestH3; 3, 3’pug-Bst; 4, Test1; 5: rab73’; and 6: break3’ (5’-cgcgatgtgttcactttgct-3’). The sequences of the other four primers have been presented previously [1].

## Results

### The Effect of Chromosomal Position on the *Pug* Phenotype

Independent insertions of a *pug*^*D*^ transgene carried within a *P* element produce varied eye pigment phenotypes, differing primarily in the diameter of the central region that lacks pteridines. This suggests that chromosomal position effects influence the expression of *pug*^*D*^. However, *P* element transposition can lead to internal deletions of the *P* element, especially when the element contains direct repeats [18,19]. Since the *pug*^*D*^ gene contains ~140 tandem repeats of AGAGAGA we suspected that transposing a *P* element carrying *pug*^*D*^ might generate deletions within the AGAGAGA repeats at a high frequency.

To test the effects of varying chromosomal position on expression of the pug phenotype without altering the AGAGAGA repeats, we moved a *pug*^D^ transgene (called 4A) to 10 target sites along chromosome *3* using FLP-mediated DNA mobilization [17]. We recovered at least one integration event at each site, with multiple independent integration events recovered for most sites. Mobilization frequencies to each *RS3r* site ranged from 1.1% to 7.5%, and are reported in Table 1.

**Table 1:** *RS3r* sites and mobilization frequencies.

	Location^a^	Integration frequency^b^
RS3r-29	*3L*, 64A, 7.8 m.u.	2.8% (2/71)
RS3r-22B	*3L*, 65F, 22.8 m.u.	2.3% (2/86)
RS3r-23	*3L*, 68C, 36.9 m.u.	3.8% (3/78)
RS3r-2	*3L*, 75C-D, 45.0 m.u.	3.4% (3/87)
RS3r-35	*3L*, 77A, 46.6 m.u.	1.1% (1/88)
Centromere	80D-81F, 47 m.u.	
RS3r-3	*3R*, 82C, 47.1 m.u.	3.8% (3/80)
RS3r-30	*3R*, 84A, 47.5 m.u.	3.8% (4/106)
RS3r-41	*3R*, 85E, 49.2 m.u.	6.3% (5/79)
RS3r-20B	*3R*, 94A, 75.6 m.u.	4.1% (4/98)
RS3r-25	*3R*, 100C, 105 m.u.	7.6% (6/79)

^*a*^ For each location a chromosome arm is indicated (except the centromere), which is followed by a cytological location and a map location in map units (m.u.). The locations of *RS3r-2* and *RS3r-3* were determined by cytology. The locations of the other eight *RS3r* sites were estimated by recombination tests based on maps in Lindsley and Zimm [10].

^*b*^ The integration frequency of a *pug*^*D*^ transgene to an *RS3r* site was measured as the percentage of males that produced at least one at least one offspring with a target site insertion.

**Fig 1:**
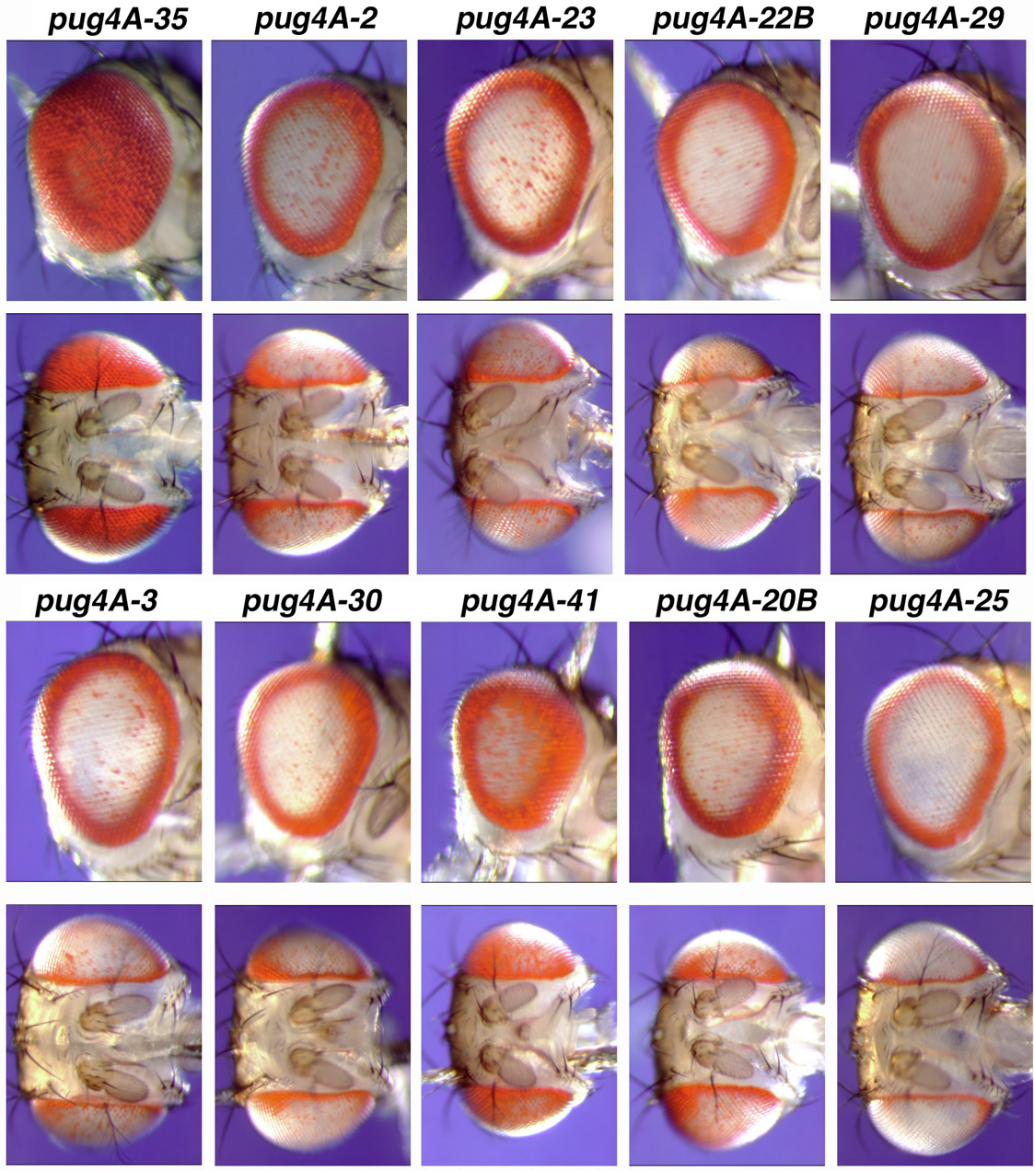
The range of phenotypes produced by FLP-mediated mobilization of *pug4A* to different target sites on chromosome *3*. All flies were *v* mutants.

Since FLP carries out reciprocal conservative recombination between *FRT*s without the participation of any host DNA exchange or repair factors, the *pug*^D^ transgene should be unchanged by the process of mobilization, and any variation in phenotype should reflect chromosomal position effects. The *pug*^*D*^ phenotype was strong at most *RS3r* sites, but did show some variation (Fig 1), having the strongest phenotype at *RS3r-25* and the weakest at *RS3r-35*. Within any line, there is little variation in phenotype and the pictures provided are highly representative of all individuals within that line. Five independent *pug4A-25* events and one *pug4A-35* event were chosen for examination by Southern blotting. The size of the *pug* transgene in these six mobilized lines was unchanged (not shown). In addition, independent integration events of the 4A *pug*^*D*^ donor at the same target site all showed identical pug phenotypes (not shown). Since the size of the *pug*^*D*^ transgene is the same at all sites, we conclude that chromosomal position can strongly modify the phenotypic expression of a *pug*^*D*^ transgene.

### The Diameter of the Central Region that Lacks Pteridines Varies with *pug*^*D*^ Copy Number

**Fig 2:**
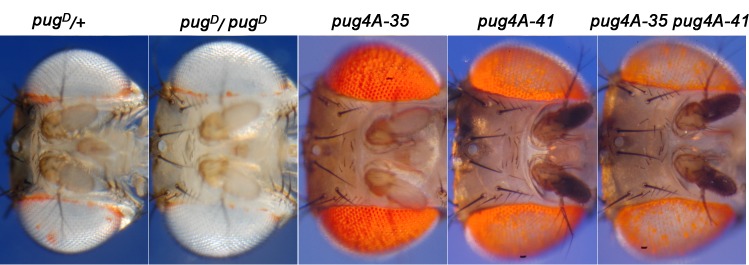
The effect of *pug*^*D*^ dosage variation on thickness of the pigmented ring. All flies were *v* mutants. Flies that were either heterozygous or homozygous for the original *pug*^*D*^ mutation are shown in the first two columns; these flies were also *karmoisin* mutants. Flies that carried one or two *pug*^*D*^ transgenes are shown in the last three columns.

The original *pug*^*D*^ mutation was not viable in homozygous condition, but since it arose on a chromosome also carrying the recessive lethal *Stubble* mutation it was not known whether *pug*^*D*^ itself was viable in homozygous condition. When we recombined *Stubble* away from the *pug*^*D*^ chromosome we found that *pug*^*D*^*/pug*^*D*^ homozygotes do occasionally survive, though at a rate well below Mendelian expectation. They exhibit even less pigment than *pug*^*D*^*/+* flies, lacking pteridine pigment entirely in several places around the periphery of the eye (Fig 2). This suggested that quantitative variation in *pug*^*D*^ might be manifest as variation in the diameter of the pteridine-lacking central region of the eye. To test this, we combined the *pug4A-35* and *pug4A-41* insertions. Together, these two transgenes exhibited a greater reduction in pteridine pigmentation than either transgene alone, approaching the phenotype produced by the original *pug*^*D*^ mutation (Fig 2). Therefore, the diameter of the central zone that lacks pterdines is a quantitative reflection of *pug*^*D*^ expression.

### Variation in AGAGAGA Repeat Length Causes Variation in *pug*^*D*^ Phenotype

This suggests that some of the variation in phenotype produced by random transgene insertions could result from variation in the AGAGAGA repeats.

We previously showed that a *pug* transgene with only 300 bp of AGAGAGA repeats was ineffective at producing the *pug*^*D*^ phenotype [1].To test this, we made use of *P* element transposition, and its tendency to alter internal repeated segments [18,19], to produce transgene insertions with varied phenotypes and potentially varied AGAGAGA repeat length.

The *P[X97*, *pug*^*D*^*]*1A, marked by *v*^*+*^, was transposed from the *X* chromosome to autosomal sites with the Δ2-3(99B) transposase source [16]. We established lines of 23 independent insertions on chromosome *2*, and 24 on chromosome *3*, for further analyses. The majority of the *v*^*+*^ male offspring also had normal pigmentation; that is, they were pug^+^. The loss of the *pug*^*D*^ phenotype in the transposed copies could be a result of a change in the *pug*^*D*^ transgene, or it might reflect chromosomal position effects, or both. To eliminate the influence of variable position effects we used FLP to mobilize sixteen of these elements, representing a broad range of phenotypes, to the *RS3r-2* site at 75C-D. We also mobilized a *pug*^*S*^ transgene, which has only 300 bp of AGAGAGA repeats [1], to this site. All were examined in a *v* background to eliminate ommochrome pigmentation.

**Fig 3:**
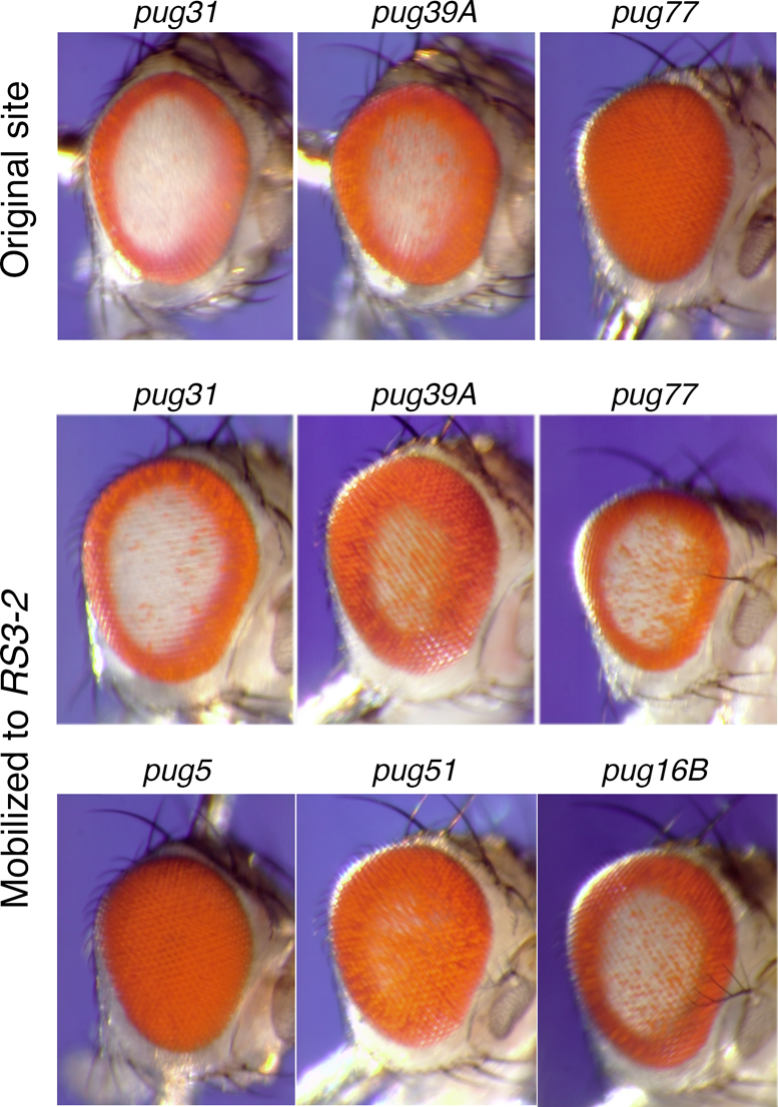
Phenotypic variation resulting from *pug*^*D*^ sequence variation. Different *pug*^*D*^ transgenes were mobilized with FLP to the *RS3r-2* target site on *3L*. The phenotypes of three of the original insertions are shown in the top row. Their phenotypes after being mobilized to *RS3r-2* are shown in the second row. The third row shows the phenotypes of three additional genes after mobilization to the same site. All flies were *v* mutants.

When FLP was used to move independent *pug* insertions to the *RS3r-2* target site we observed a wide range of pigmentation patterns in the eye, indicating that new variants had arisen among the transposed *pug* genes (Fig 3 and others not shown). The phenotypes range from completely normal (*pug*^*s*^*-2*) to eyes that are similar to the original *pug*^*D*^ (*pug4A-2*; Fig 1). We also observed that the phenotype of a *pug*^*D*^ transgene could change substantially upon mobilization to *RS3r-2*. In some cases, transgenes that were nearly *pug*^*+*^ at their original site expressed a strong *pug*^*D*^ phenotype at the *RS3r-2* site (for instance, *pug77*), or vice-versa (*pug39A*; Fig 3). As mentioned above, there was little variation within any particular line.

**Fig 4:**
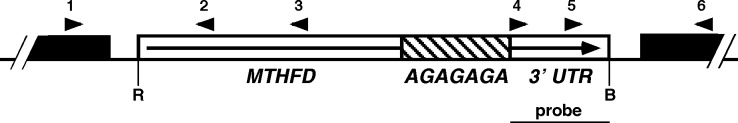
The structure of *pug*^*D*^ in the vector *P[X97]*. The 3.4 kb *Eco*RI-*Bam*HI (R, B) fragment of *pug*^*D*^ includes three parts: the 5’ portion from *pug*^*+*^ (*MTHFD*); the one kb AGAGAGA repeat; and, the putative 3’ UTR from the *rab7* region. The arrow indicates the direction of transcription. The solid boxes to either side represent sequences from the *white* gene of the vector. The probe used for Southern analyses is indicated. Arrow heads represent primers used for PCR. See Materials and Methods for details.

**Fig 5:**
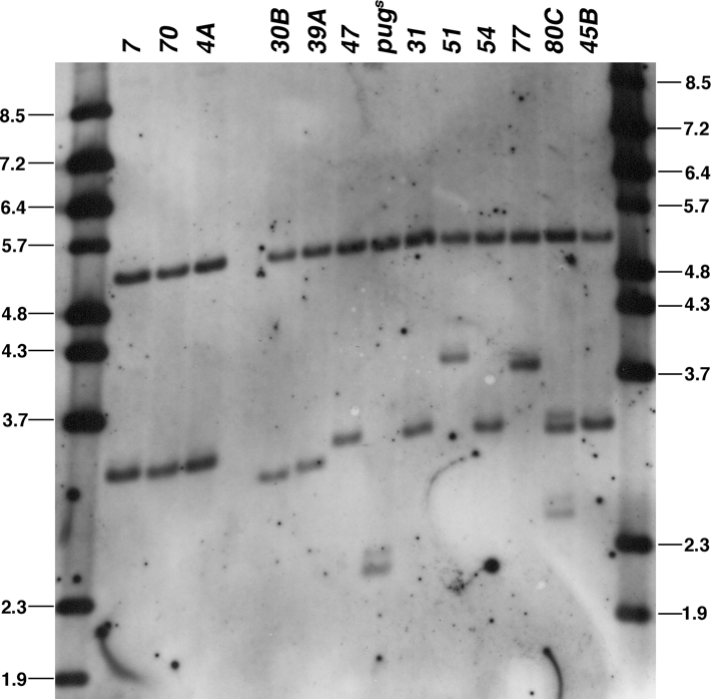
Characterization of *pug*^*D*^ transgenes by Southern blotting. Genomic DNA was prepared from flies hemizygous for *pug* transgenes after they were mobilized to the *RS3r*-2 target site. DNA was digested with *Eco*RI and *Bam*HI and probed with the fragment indicated in Fig 4. Molecular weight markers are shown in the left and right lanes, with sizes indicated in kb. The *pug* transgene line number is indicated for each lane, *e*.*g*., the first lane is *pug7-2*. The 5 kb band in all sample lanes is the endogenous *rab7* band.

Molecular analysis of the variant *pug* genes showed that AGAGAGA repeat lengths that differed substantially from the ~1.0 kb found in the original *pug*^*D*^ allele resulted in a reduced phenotype. Fig 4 depicts the structure of the *pug*^*D*^ region after mobilization to the *RS3r* target site. We deduced the length of the repetitive stretch by first using Southern blots to measure the size of the *Eco*RI—*Bam*HI fragment. A sample of these data are shown in Fig 5. PCR analyses were then done to measure the sizes of the regions upstream and downstream of the repeats, using primer pairs 1,2 and 1,3 upstream and 4,6 and 5,6 downstream (locations diagramed in Fig 4; results not shown). With this combination we can estimate the length of the region between primers 3 and 4, which consists mostly of the AGAGAGA repeats. Any change in this region is attributed to a change in the length of the AGAGAGA repeats. (It was not possible to simply use PCR with spanning primers to determine the length of the AGAGAGA repetitive section because we were unable to perform PCR across such repeats that are longer than 300 bp.) The estimated AGAGAGA repeat length for each insertion is given in Table 2.

**Table 2:** *P* element lines for mobilization to *RS3r-2*.

Line	Chromosomal location	Origin	Pigmented progeny	Length of the AGAGAGA repeat^c^
4A	2	Injection	+	~1000 bp
7	2	Transposition	+	~1000 bp
16B	2	Transposition	+	~800 bp
27C	3	Transposition	+	~1000 bp
30B	2	Transposition	+	~600 bp
31	2	Transposition	+	~1000 bp
39A	2	Transposition	+	~650 bp
45B	3	Transposition	+	~1000 bp
46	2	Transposition	+^a^	ND
47	3	Transposition	+	~950–1000 bp
50	3	Transposition	-^b^	ND
51	3	Transposition	+	~1400 bp
54	3	Transposition	+	~1000 bp
70	3	Transposition	+	~1000 bp
74	3	Transposition	-^b^	ND
77	2	Transposition	+	~1300 bp
80C	3	Transposition	+	~1000 bp
*pug*^*s*^	2	Injection	+	~300 bp

^*a*^ The pigmented progeny may be the result of an illegitimate integration event (see RESULTS).

^*b*^: A somatic assay did not reveal successful integration in the eye. Not characterized further. (see RESULTS).

^*c*^: The lengths of the GAGA repeat were estimated by Southern blot and PCR analyses (see RESULTS). ND, not done.

The *pug4A* transgene appears to have ~1 kb of AGAGAGA repeats, the same repeat length as the original *pug*^*D*^ allele. It has a strong pug^D^ phenotype at most, though not all, sites, and serves as one standard for comparison (Fig 1). The other standard is the *pug*^*S*^ gene, carrying 300 bp of AGAGAGA repeats, derived by loss of a portion of the repetitive sequence during growth in bacteria [1]. Judged by Southern blotting, lines 4A, 7, 31, 45B, 47, 54, and 70 all have the full length repeats. However, PCR analysis with primers 4, 6 and 5,6 indicated that a non-repeat portion of *pug47* is slightly larger than the others (not shown), meaning that its repetitive portion must be slightly shorter to produce an *Eco*RI—*Bam*HI fragment of the same size. However, the difference is small, perhaps 50–100 bp, and our Southern blot is probably not capable of indicating whether there is such a small difference in the size of the *Eco*RI—*Bam*HI band. These lines, and others that have approximately the same length of repeats as *pug4A*, produced a phenotype that was indistinguishable from *pug4A* when inserted at the *RS3r-2* target site (Table 2; Fig 1).

In contrast, the lines with clear alterations in repeat length, both longer and shorter, have weaker phenotypes (Fig 3). The *pug51* and *pug77* genes both have repeat segments longer than the original *pug*^*D*^ allele, yet produce weaker phenotypes: especially *pug51*, which has the longest repeat segment of ~1400 bp. Four of the alleles, *16B*, *30B*, *39A* and *pug*^*s*^, have AGAGAGA repeats that are significantly shorter than the 1 kb found in *pug*^*D*^, and they all have a reduced phenotype. Among these four shortened alleles, *16B* has the longest repeat length (~800 bp) and produces the strongest phenotype of that group; *pug*^*s*^ has the shortest repeat (300 bp), and produces the weakest phenotype, which is essentially pug^+^. Finally, the *pug80C* transgene appears to have a complex rearrangement, which we did not completely characterize, and it produces a strong phenotype. The correlation between repeat length and phenotype was not perfect: *pug30B* has a slightly shorter repeat than *pug39A* (600 bp *versus* 650 bp), yet, the former produces a slightly stronger phenotype (not shown). It is possible that variation in the sequence of the non-repetitive portion may account for such minor differences. Notwithstanding this particular example, the overall conclusion is that the strength of *pug*^*D*^ alleles increases as the repeat length increases from ~300 bp, reaching its strongest effect at a length of ~ 1000 bp, and then decreasing as the repeat length extends beyond ~1000 bp. The variation in phenotype was similar to that which could be produced by altering *pug*^*D*^ transgene dosage, indicating that repeat length variants effect a quantitative, not qualitative, reduction in the effect of the *pug*^*D*^ alleles.

### The Ring Pattern of Pigment in *pug*^*D*^

We previously proposed a model in which the pattern of *pug*^*D*^ pigmentation is determined by the architecture of the compound eye (discussed further below). However, *pug*^*D*^ mosaics can be observed, as a consequence of position effect variegation [1], or by FLP-mediated mosaicism (results not shown). This indicates that the action of *pug*^*D*^ is cell autonomous, at least to a large degree, and suggests an alternative model: that the ring of pigment in *pug*^*D*^ could result from repression of *pug*^*D*^ expression in cells at the periphery of the eye.

A prominent candidate for regulating such expression is *wingless* (*wg*). Tomlinson [11] summarized several features that distinguish the periphery of the eye from the center: the most interesting aspect with respect to *pug* is that there are pigment cells surrounding the eye which are not associated with photoreceptors. Tomlinson called this the Pigment Rim (PR). Cells of the PR apparently correspond to the cells that retain pteridine pigmentation in a *v; pug*^*D*^*/+* fly [1]. Formation of the PR results from the death of photoreceptor cells in the peripheral ommatidia, and this death is directed by *wg* expression [11,14]. The expression pattern of *wg* is highly reminiscent of the pigment pattern seen in *v; pug*^*D*^*/+* flies, with expression in the adult head limited, for the most part, to cells of the head capsule that surround the eye (see Fig 2D in Tomlinson [11]). These cells are directly in contact with the PR cells. Thus, *wg* is a strong candidate for controlling the expression of *pug*^*D*^.

**Fig 6:**
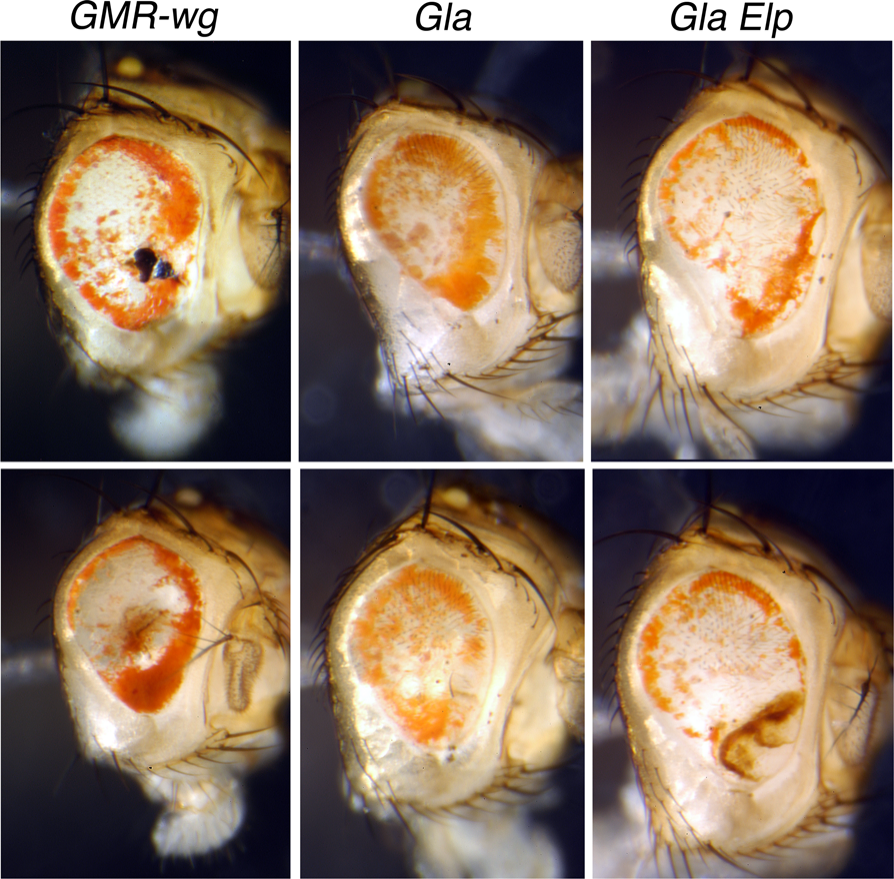
The effect of ubiquitous *wingless* expression of *pug*^*D*^ pigmentation. All eyes are from *v; pug*^*D*^*/+* males that also carry: a *P* element expressing *wg* under the *glass multiple repeat* enhancer (*GMR-wg*); the *Glazed* mutation alone (*Gla*^*1*^*/+*); or *Gla*^*1*^ and *Ellipse* (*Gla*^*1*^*/+ Elp/+*). Typical phenotypes are shown.

To test whether *pug*^*D*^ responds to *wg* we generated flies carrying *pug*^*D*^ and *GMR-wg*, a *P* element construct that expresses *wg* throughout the eye during late development. The effect of this expression is to cause the death of photoreceptor cells throughout the eye, producing an adult eye that is composed almost entirely of pigment cells, much like the PR of a normal eye [11]. If *pug*^*D*^ is repressed by *wg*, we expect that the ubiquitous *wg* expression in the eye produced by *GMR-wg* should repress *pug*^*D*^ throughout the eye, allowing pteridine pigmentation throughout. This was not seen (Fig 6). Instead, the *pug*^*D*^ ring of pigment was still apparent. The complete absence of bristles in these eyes confirms that *wg* was expressed throughout the eye. We also combined *pug*^*D*^ with the mutation *Gla*^*1*^, a dominant allele of *wg* with expression throughout the eye, much like *GMR-wg* [12], and still saw the *pug*^D^ ring phenotype (Fig 6). Finally, a *sev-wg* construct [14] was tested with the same results (not shown). The expression of *wg* throughout the eye did not suppress the pug^D^ phenotype in any case. We conclude that the pug^D^ phenotype is not a response to *wg* signalling.

There was some expansion of the pigmented region in some of the double mutant individuals. We suspect this is a consequence of disruption of the structure of the eye, and not a specific repression of *pug*^*D*^, because similar results can be produced by injury to the eye (see below).

Though we did not count offspring, the *Gla*^*1*^ *pug*^*D*^ or *GMR-wg pug*^*D*^ double mutants were quite rare in our crosses. Many pharate adults were produced, and when dissected from their pupal cases, they were found to be the missing double mutants. These pharates appeared to be fully differentiated adults, but were extremely dehydrated, usually having completely flattened abdomens. A common characteristic of these flies was that at least one of the eyes had a large black scab-like mass on its surface. Evidence of this may be seen in the infrequent survivors, for example the fly shown at the upper left of Fig 6. We suspect that the cause of lethality in the double mutants is that the eye bursts from turgor pressure, allowing internal fluids to leak out and cause lethal dehydration. We have previously noted that the eyes of *pug*^*D*^ flies seem somewhat fragile [1]. In combination with *Gla*^*1*^, the integrity of the eye is apparently compromised to such a degree that it is often lethal.

**Fig 7:**
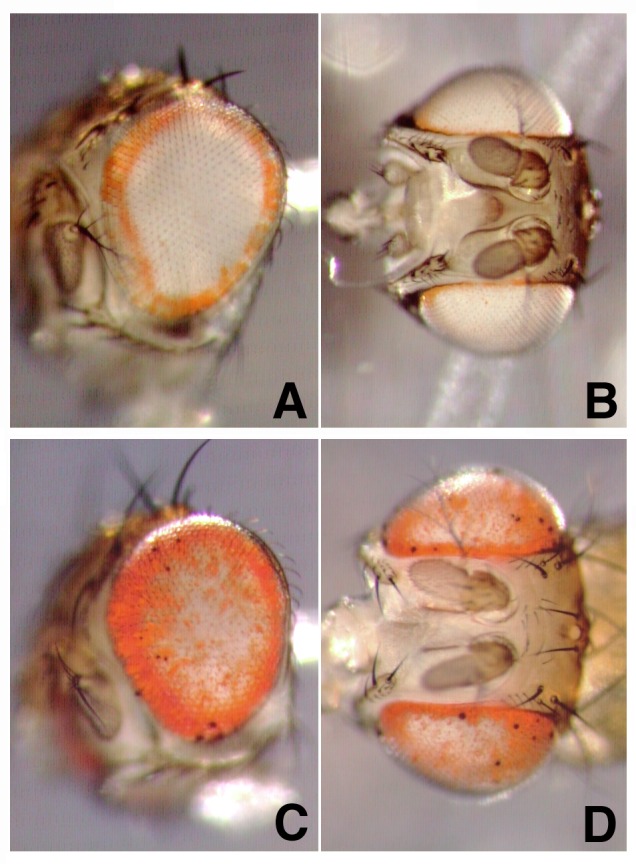
The effect of baculovirus P35 expression on *pug*^*D*^. A, B: eyes from *v; pug*^*D*^*/+* flies. C, D: eyes from *v; pug*^*D*^ */P[GMR-P35]* flies.

We also carried out the reciprocal test of preventing photoreceptor cell death by using *GMR-P35*, a construct that expresses the anti-apoptotic P35 protein from baculovirus throughout the eye and blocks virtually all cell death [13,20]. The ring of pigment was still evident (Fig 7). In fact, the *pug*^*D*^ *GMR-P35* flies even show an increase in cells with pigment. This is likely the result of disrupting the regular ommatidial structure in these eyes (see below).

Although *wg* did not affect *pug*^*D*^, there may yet be some relationship between *wg* and pteridine pigment patterning. We noticed that in *v; Gla/+* or *v; GMR-wg/+*, there is a small region of the eye, typically in the ventral portion, that is unpigmented. It is not simply the case that this portion of the eye is devoid of pigmented cells, because in a *v/v*^*+*^*; Gla/+* or *v/v*^*+*^*; GMR-wg/+* fly this part of the eye is pigmented, though it is clearly a different color than the remainder of the eye, no doubt reflecting the loss of pteridine but not ommochrome pigmentation in this region (not shown).

### Eye Pigmentation Can Be Partially Restored by Direct Eye Injection

Previously, we proposed the model that pigment cells at the periphery of the eye are able to take up purines from neighboring non-eye tissue, providing them with a critical precursor for pigment synthesis, and that *pug*^*D*^ interferes with transport to more interior cells of the eye.

**Fig 8:**
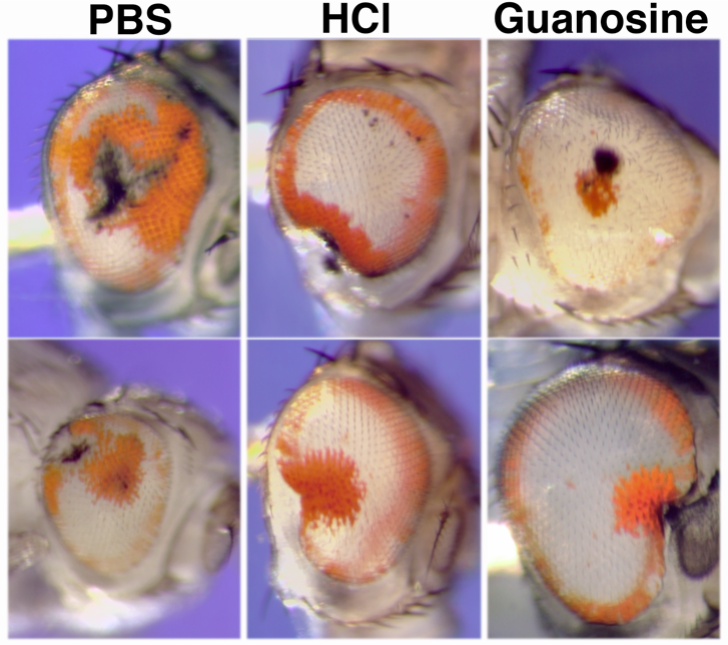
Extra pigmentation produced by injury to pupal eyes. All flies have a genotype of *v; pug*^*D*^ */TM3*. Typical examples of eyes injected with Phosphate-buffered saline (PBS, left column), water acidified with hydrochloric acid (HCl, middle column), or an acidic guanosine solution (Guanosine, right column) are shown. In all cases some additional regions of pigmentation are seen. The site of injury produced by needle entry can sometimes be clearly seen by the black scar that is produced.

To test this, we designed an experiment to deliver purines directly to cells in the center of the eye by injection into the eyes of early pupae. It has been shown that pupal eyes incubated *in vitro* can take up guanine derivatives for pigment synthesis [21]. We predicted that if we could supply purine precursors to the center of the eye by injection, we might be able to restore pigmentation. We previously determined that the developmental period in which *pug*^*D*^ is most effective in pigment elimination is during the first two days of pupal development [1]. The best time to restore pigmentation by injection is likely to be within these 2 days. We injected the eyes of *v; pug*^*D*^ */+* pupae at stages ranging from 0 to 2 days into pupation with solutions of either guanosine, PBS, or an acidified aqueous solution. For all three solutions, areas of extra pigmentation in the eye were often observed (Fig 8).

These experiments show that the center of a *pug*^*D*^ eye still has the ability to make pteridine pigment. This is consistent with the conclusion that *pug*^*D*^ does not eliminate pigmentation by killing pigment cells. It is unlikely that a common chemical compound in all three solutions has been provided by the injection which allowed the synthesis of pteridines around the injected areas. Instead, the occurrence of injury to the eye seems to be sufficient to produce pigment in new areas of the eye.

## Discussion

The variation that we observed with various *pug*^*D*^ transgenes is most obviously a variation in thickness of the pteridine-pigmented ring with a complementary variation in the size of the central region that lacks pteridine pigmentation. A similar effect can be produced by varying *pug*^*D*^ gene copy number. In other words, the size of the unpigmented central zone is a quantitative manifestation of the *pug*^*D*^ phenotype. Our experiments show that chromosomal position effects are responsible for some of this variation. These results are consistent with previous findings that different chromosomal insertion sites have mostly quantitative effects on the expression of transgenes (e.g., [22,23]).

A second strong influence on the *pug*^*D*^ phenotype is the length of the AGAGAGA repeats, which also produces variation in the thickness of the pigmented ring. Phenotypic severity is not strictly correlated with the length of AGAGAGA repeats: repeated segments that are either shorter or longer than the ~1 kb found in the original *pug*^*D*^ allele can reduce the size of the central region that lacks pteridine pigmentation. Although we have shown that translation of the repeats is necessary for the pug phenotype [1], our results do not speak to the mechanism by which the repeat variation leads to phenotypic variation. The varied AGAGAGA repeats might affect expression of the gene, the stability of the mRNA or the protein, or the efficacy of its action. The fact that this sequence variation produces the same phenotypic effect as variance in gene copy number suggests that, whatever the molecular mechanism, the effect is essentially quantitative. It will be of great interest to determine whether the *pug*^*D*^ mode of action shares any commonalities with human diseases that arise from the translation of lengthy monotonic amino acid segments [24].

We previously discussed the possibility that the scattered spots of pigment seen in the center of a *v; pug*^*D*^*/+* eye are a reflection of classical position effect variegation (PEV; [25]) resulting from the presence of the heterochromatic AGAGAGA repeats [1]. However, the *pug*^*D*^ phenotype is unaffected by a suppressor of PEV (*Su(var)205*) or by a PEV-enhancing genotype (*X0* males; our unpublished results). It seems unlikely that the variegated phenotype is a result of PEV. There does seem to be a correlation between the frequency of spots in the center of the eye and the thickness of the pigment ring (Fig 1). Thus, the absence or appearance of spots appear to be another manifestation of the strength or weakness of the phenotype.

Obtaining these results depended on our ability to alter one of two factors in isolation: either gene sequence or chromosomal position. The *pug*^*D*^ gene sequence was altered by transposase, or by the intrinsic instability of this sequence in bacteria, and the altered genes were then all placed at the identical genomic site by FLP-mediated DNA mobilization [17]. Conversely, chromosomal position effects were tested by using this method to move a single allele to multiple sites. Several other methods that use site-specific recombination to integrate different genes at specified target in Drosophila have been produced. The method of transgene co-placement, based on the cre-*loxP* site-specific recombination system, was developed to compare the effects of two genes at the same site and eliminate uncontrolled variability resulting from position effect [26,27]. More recently, the integrase of bacteriophage ØC31 [28], the FLP recombinase [29] and the Cre recombinase [30] have been used in *Drosophila* to integrate transgenic DNA at a specific site directly upon injection. The ØC31 integrase method is efficient, stocks of flies with different chromosomal *attP* sites for integration are available, and it has therefore gained wide acceptance for transformation to a specific site [31]. However, we still see a unique advantage to our approach in this particular case—by generating an initial transgene inside a *P* element we were able to use transposase to generate internal sequence variants, which showed that the AGAGAGA repeat length affects the phenotype. Additionally, a very large collection of precisely localized *P[RS3]* insertions that can serve as target sites has been produced [32].

Most genes that contribute to eye color have mutant phenotypes that affect pigmentation uniformly across the eye. *pug*^*D*^ is a rare example in which the eye color defect is patterned. The fact that *pug*^*D*^ variation appears as variation in the thickness of the peripheral pigmented ring suggests the presence of a factor that originates in the periphery and spreads inward. We have considered two classes of model for what this factor might be and how it could work. One possibility is that a signaling molecule may emanate from outside the eye and act to repress the expression of *pug*^*D*^. Our experiments show that wingless is not this molecule. It is still possible that *pug*^*D*^ is negatively regulated by some peripheral signal that is unrelated to *wg* and has yet to be identified. Alternatively, *pug*^*D*^ may be responding positively to a signal that emanates from the center of the eye. However, since no particular distinctive feature of the eye center has been identified we consider this unlikely. Furthermore, our finding that pigmentation can be rescued simply by injury to the pupal eye does not support models involving such signalling.

The model we favor supposes that *pug*^*D*^ creates a shortage of some metabolite required for pigment formation, but that this shortage can be overcome by uptake of some still unidentified compound. To account for the ring pattern of pigmentation, this compound would be taken up only by cells around the periphery of the eye, but it may be transported to more interior cells if it is present in excess. In *pug*^*D*^*/+* flies cells around the edge of the eye may import enough of the critical substance for their own use, accounting for the ring of pigment. If expression of *pug*^*D*^ is reduced then this substance may be transported from cell to cell further towards the center of the eye until it is no longer in surplus. This hypothesis requires that all cells in the eye are capable of transport of this compound, but only cells around the periphery are capable of taking it up from outside of the eye. One way to achieve such a limitation would be if the transport machinery were localized along the elongated surfaces of the secondary pigment cells that produce pteridine pigment, but not at the bases of those cells (represented diagrammatically in [1]). Localized transport of the rosy protein (xanthine dehydrogenase) has been shown, but it occurs instead at the base of pigment cells [33,34]. We found that physical injury to the eye was sufficient to produce new regions of pigmentation. This observation is consistent with our hypothesis. Cell surfaces that would normally be in contact only with other cells of the eye may be exposed by the injury, allowing uptake of the critical compound in the new location. The increased number of internal cells with pigment in the *P35* and *wingless* expression experiments may well result from the disruption of ommatidial structure in those genotypes, allowing transport to internal cells.

The identification of the transported compound might go a long way to help understand the mechanism of *pug*^*D*^ action. One possibility is that the Pug^D^ protein binds and sequesters the tetrahydrofolate substrate (MTHF) that is used as a cofactor in purine biosynthesis. The import of either folate or purines to the eye by cells around the periphery could restore pigmentation and explain the ring pattern of pigmentation produced by *pug*^*D*^.

If our model is correct, one might expect to see reduced health or viability of *pug*^*D*^ flies, since mutants affecting *de novo* purine synthesis have reduced viability [35,36]. There probably is a reduction in health of cells of the eye, since the eyes of *pug*^*D*^*/+* flies to appear to be somewhat fragile [1]. Both *pug*^*D*^ and the reduced dosage of *pug*^*+*^ in these heterozygotes could be contributing factors to this effect. This impairment is especially evident in the *Gla*^*1*^*/+; pug*^*D*^*/+* double mutant combination which is frequently lethal, though neither mutation by itself causes significant lethality. Cells in the eye finish their program of cell division around the time of pupariation and are likely to be particularly stressed because of the need for large quantities of purines to make pigment, which occurs after the larvae have stopped feeding [7]. It is conceivable that these cells have a sufficient store of purines to undergo metamorphosis, but not enough to carry out the more demanding task of producing pigment. For this they would need to synthesize additional purines, or transport purines or other precursors from outside.
